# The reaction mechanism of the *Ideonella sakaiensis* PETase enzyme

**DOI:** 10.1038/s42004-024-01154-x

**Published:** 2024-03-27

**Authors:** Tucker Burgin, Benjamin C. Pollard, Brandon C. Knott, Heather B. Mayes, Michael F. Crowley, John E. McGeehan, Gregg T. Beckham, H. Lee Woodcock

**Affiliations:** 1 Department of Chemical Engineering, University of Washington, Seattle, WA USA; 2 Thayer School of Engineering, Dartmouth College, Hanover, NH USA; 3 Renewable Resources and Enabling Sciences Center, National Renewable Energy Laboratory, Golden, CO USA; 4 Department of Chemistry, University of South Florida, Tampa, FL USA; 5 Centre for Enzyme Innovation and Institute for Biological and Biomedical Sciences, University of Portsmouth, Portsmouth, UK

**Keywords:** Enzyme mechanisms, Computational chemistry, Hydrolases, Reaction mechanisms

## Abstract

Polyethylene terephthalate (PET), the most abundantly produced polyester plastic, can be depolymerized by the *Ideonella sakaiensis* PETase enzyme. Based on multiple PETase crystal structures, the reaction has been proposed to proceed via a two-step serine hydrolase mechanism mediated by a serine-histidine-aspartate catalytic triad. To elucidate the multi-step PETase catalytic mechanism, we use transition path sampling and likelihood maximization to identify optimal reaction coordinates for the PETase enzyme. We predict that deacylation is likely rate-limiting, and the reaction coordinates for both steps include elements describing nucleophilic attack, ester bond cleavage, and the “moving-histidine” mechanism. We find that the flexibility of Trp185 promotes the reaction, providing an explanation for decreased activity observed in mutations that restrict Trp185 motion. Overall, this study uses unbiased computational approaches to reveal the detailed reaction mechanism necessary for further engineering of an important class of enzymes for plastics bioconversion.

## Abstract (web summary)

Polyethylene terephthalate (PET) can be depolymerized by the *Ideonella sakaiensis* PETase enzyme, however, questions remain about the precise catalytic mechanism. Here, the authors use unbiased QM/MM MD simulations to determine optimal mechanistic descriptions of the acylation and deacylation reactions, revealing the rate-limiting step and key interactions within the catalytic triad and Trp185 conformation.

## Introduction

In 2016, Yoshida et al. reported the discovery of a soil bacterium, *Ideonella sakaiensis* 201-F6^1^, that secretes a two-enzyme system for the extracellular depolymerization of poly(ethylene terephthalate) (PET). PET is the most prevalent polyester manufactured today and a major plastic pollutant in the environment. In the two-enzyme system, PETase acts directly on the solid PET plastic to liberate bis-hydroxyethyl terephthalate (BHET) and mono-hydroxyethyl terephthalate (MHET), wherein MHET is further cleaved into ethylene glycol and terephthalic acid by the MHETase enzyme, providing substrates for *I. sakaiensis* catabolism^1–4^. *Is*PETase is one of several microbial polyester hydrolases with predicted^5,6^ and demonstrated^5–9^ ability to depolymerize PET. Several successful engineering efforts of PETase, MHETase, and related serine hydrolases have resulted from a combination of rational engineering, directed evolution, and advanced computational strategies^3,4,10–17^, and some of these have demonstrated that increased enzyme melting temperature can lead to improved PET hydrolysis activity^11,14,15,18^.

**Fig. 1:**
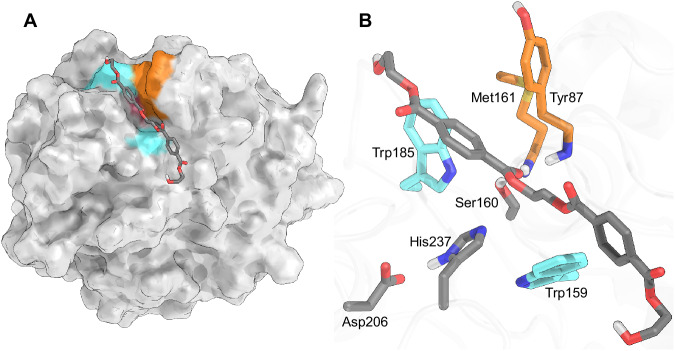
The *Ideonella sakaiensis* PETase enzyme and active site. **A** Surface depiction of the equilibrated Michaelis complex. PETase (white surface) bound to the PET dimer (gray sticks), with catalytic Ser160 (red surface), Trp residues (teal surface), and oxyanion residues (orange surface) highlighted. **B** Detailed view of the active site, showing the catalytic triad (Ser160, His237, and Asp206) and PET dimer as gray sticks, Trp185 and Trp159 as teal sticks, and the oxyanion hole residues, Met161 and Tyr87 as orange sticks. PET is oriented with respect to Ser160 to allow for nucleophilic attack of the PET carboxyl carbon. Hydrogen bonds are formed between the catalytic residues, as well as between the carboxyl oxygen of PET and the oxyanion hole. Trp185 and Trp159 interact with the PET aromatic rings through a parallel-displaced and an edge-to-face π-π interaction, respectively. For clarity, non-polar hydrogens are not shown.

The active site of PETase (Fig. 1) clearly indicates that it is a serine hydrolase, with the canonical serine-histidine-aspartate (or glutamate in some cases) catalytic triad, suggesting that PETase follows a two-step acylation and deacylation mechanism^19,20^. Ligand-bound crystal structures and docking results from PETase structural studies have guided speculation about the reaction mechanism, with conflicting hypotheses regarding the interaction between Trp185 and the substrate (numbering based on the structure published by Austin et al.)^10^. Reported crystal structures assign the side chain of Trp185 to three different conformations with molecular dynamics (MD) simulations showing that Trp185 is highly flexible^10,21,22^. Flexible docking results from Austin et al. suggest that Trp185 adopts conformations different than those found in the crystal structures, finding a parallel-displaced π–π interaction between PET and Trp185 in wild-type PETase, and an edge-to-face π–π interaction (see Wheeler and Bloom^23^ for examples) in a double-mutant PETase^10,23^. Joo et al. reported covalent docking results wherein PET and Trp185 are oriented in what appears to be a parallel-displaced π–π interaction^24^. A hypothesis that emerged from Guo and coworkers based on bound substrate and product analogs is that PET adopts an edge-to-face (described as T-shaped) π–π interaction with Trp185 for the reaction^21,25^. Following the reaction, the authors propose that the product rotates to adopt a parallel-displaced (described as face-to-face) π–π interaction with Trp185 that aids the leaving group’s departure from the active site.

In serine hydrolases, more broadly, conflicting hypotheses have been presented regarding the mechanism of proton transfer via the histidine of the catalytic triad^19,26–28^. An NMR study of subtilisin suggested that the reaction used a “flipping histidine” mechanism (Fig. S1), wherein N_ε2_ of histidine accepts a proton from the nucleophile, followed by a 180° rotation of the imidazole ring and subsequent delivery of the proton from the N_δ1_ position to the leaving group^29^. Other studies, however, have found evidence of a “moving histidine” mechanism (Fig. S1), where the side chain of the histidine uses a lateral translational motion to “shuttle” the proton from the nucleophile to the leaving group via N_ε2_^27^. The role of this histidine has been proposed to include the interaction with the aspartate of the catalytic triad, specifically in characterizing the hydrogen bond between the two. It has been proposed that the reaction could use a double-proton transfer mechanism in a “charge-relay” (Fig. S1), wherein aspartate abstracts a proton from histidine followed by histidine abstracting a proton from the catalytic serine^19,27^. While some studies have found evidence of the double-proton transfer, other studies have found evidence against the mechanism^19^. QM/MM computational studies have undertaken to address some of these questions^30–36^ but have presented conflicting results, including the free energy barriers, which is the rate-limiting step, and whether tetrahedral geometries along the reaction pathway are metastable intermediates or transition states, leaving open questions regarding the PETase reaction mechanism.

In light of the open questions described above related to PETase action and generally in serine hydrolase mechanisms, and further to provide an accurate fundamental mechanistic understanding for future enzyme engineering efforts, here we seek to elucidate the detailed PETase reaction mechanism using unbiased quantum mechanical/molecular mechanical (QM/MM) MD simulations^37^. Specifically, we use transition path sampling (TPS) and inertial likelihood maximization (iLMax) to determine optimal mechanistic descriptions of the acylation and deacylation reactions^38–40^. TPS was employed as it requires no a priori knowledge of the reaction mechanism, and iLMax allows for the extraction of complex reaction coordinates (RCs) with high transmission coefficients that are capable of capturing subtleties of the reaction mechanism that may not be revealed by preconstructed RCs^38,40,41^. The resulting RCs for each reaction step were validated as described below and subsequently used to compute the free energies and rate constants of the acylation and deacylation steps, enabling prediction of the rate-limiting step, free of bias from a pre-assumed reaction coordinate. These simulations also allow monitoring of interactions within the catalytic triad and the Trp185 conformation along the reaction steps, to interrogate key mechanistic questions raised from structural studies. From these simulations, we predict that PETase employs the moving histidine mechanism, and we find no evidence of proton transfer between the aspartate and histidine of the catalytic triad. Importantly, the simulations indicate that the motions of Trp185 facilitate catalysis and provide a likely molecular rationale for why mutations, such as S214H (Ser185 in the study by Guo and coworkers), restrict the motion of Trp185, decrease PETase activity^21,25^.

## Results

### Elucidating the acylation reaction coordinate and estimation of reaction rate

To set up a PETase system for QM/MM simulations, we began with a Michaelis complex of a hydroxyethyl-capped PET dimer bound to PETase from our prior docking simulations^10^. The QM/MM equilibrated Michaelis complex used for the TPS simulations is shown in Fig. 1, with the catalytic triad, Ser160, His237, and Asp206, highlighted. The nucleophilic Ser160 is activated by catalytic residues His237 and Asp206 and is oriented to attack one of the central carboxyl carbons in between the two aromatic rings of the PET dimer. The oxyanion hole, comprising the backbone amines of Met161 and Tyr87, forms a hydrogen bond to the carboxyl oxygen of PET and provides stabilization for charge accumulation during the reaction. The Trp185 and Trp159 residues that flank the catalytic triad aid in positioning the PET ligand for attack via stabilizing aromatic interactions. Following QM/MM equilibration with DFTB3^42^, a putative transition state structure was identified using the *find_ts* procedure of ATESA, an automated aimless shooting workflow^43^. Aimless shooting (AS) was then used to generate an ensemble of 13,750 unbiased trajectories, of which approximately 40.5% were reactive (i.e., connected the reactant and product states)^40^.

**Fig. 2:**
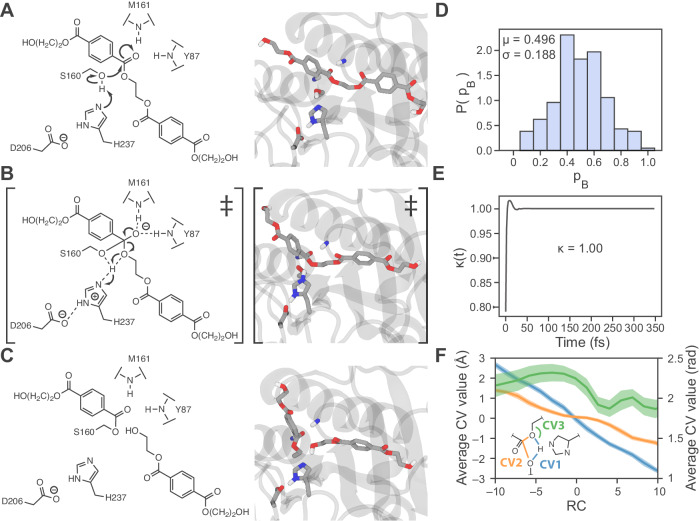
PETase acylation reaction. **A**–**C** Scheme depicting the steps of the acylation reaction with schematics (*left*) and corresponding model snapshots (*right*). (A) Michaelis complex, where the catalytic Ser160 and PET ligand are oriented for reaction. **B** TS configuration where Ser160 has attacked the carboxyl carbon of PET, Ser160 has transferred the proton to His237, the carboxyl carbon of PET adopts a tetrahedral geometry, and His237 is midway through the proton transfer. **C** Product of acylation (acyl-enzyme intermediate, AEI) where Ser160 is covalently bound to the PET fragment (i.e., MHET), the scissile ester bond has been cleaved, and the BHET product has accepted the proton from His237. **D** Committor probability (*p*_*B*_) histogram obtained from unbiased trajectories initiated from 208 putative TS structures according to the discovered acylation RC. **E** Reactive flux correlation function computed using unbiased trajectories initiated from the putative TS region. (**F**) Average collective variable (CV) values along the RC were obtained from the full ensemble of unbiased trajectories from aimless shooting. These distances are CV1 = the difference (in Å) between the distances Ser160 H—PET carboxyl O and Ser160 O–Ser160 H; CV2 = the difference (in Å) between the distances Ser160 O–PET carboxyl C and PET carboxyl C–PET carboxyl O; and CV3 = the angle (in radians) formed by Ser160 H–PET ether O–BHET C. CVs 1 and 2 correspond to the axis at left while CV3 corresponds to the axis at right. The coordinates from these trajectories were binned according to the value of the RC from −10 to +10 using 16 bins, wherein the CVs in each bin were averaged, and the standard deviation was taken as an error.

Through inspection of the reactive AS trajectories, illustrated in Fig. 2A-C, the reaction scheme proceeds through the following sequence: (1) from the reactant state, Ser160 transfers a proton to His237 and attacks the carboxyl carbon of the PET dimer; (2) the PET carboxyl carbon adopts a tetrahedral TS configuration with His237 at the midpoint of the proton shuttle; and (3) the TS collapses, cleaving the scissile ester bond, with the BHET leaving group accepting the proton from His237. The completion of this reaction step leaves the system in the acyl-enzyme intermediate (AEI) state (i.e., the product state of acylation) with a PET fragment (i.e., MHET) bonded to PETase via Ser160.

The ensemble of unbiased trajectories from AS enables the extraction of the acylation RC^38^. The iLMax method was used to extract RCs from the AS ensemble (see the section “Methods” for more details) as a linear combination of collective variables (CVs)^41^. In total, 299 CVs (automatically selected by ATESA^43^ with a cutoff distance of 5 Å from each reactive atom) were screened and included all of the distances, angles, and dihedral angles of atoms inside the cutoff radius, as well as the differences of distances between the reactive atoms. ATESA was used to select an optimized linear combination of CVs with iLMax, and then the appropriateness of the RC was confirmed with a committor probability (*p*_B_) histogram test^40,43–45^. The optimal acylation RC has the form $4.18-2.32* {{\rm {CV}}}1-2.59* {{\rm {CV}}}2-0.0119* {{\rm {CV}}}3$, based on the CV definitions shown in Fig. 2F and described in the corresponding caption. A high-quality RC should exhibit a *p*_B_ histogram peaked near 0.5, demonstrating that trajectories initiated from the putative TS region, according to the RC, have a ~50% probability of evolving to either reactants or products^40,44^. The *p*_B_ histogram for the acylation RC, which shows results from 10 simulations each for the 208 AS points with RC values nearest to 0 (i.e., the predicted transition state), has a near-ideal shape, as shown in Fig. 2D.

The acylation RC includes contributions from differences in distances that are critical to the reaction, namely those associated with bond breaking and forming during nucleophilic attack, scissile ester cleavage, and proton transfer. In addition, the RC accounts for the angle formed between the proton that transfers from Ser160 to the BHET product, the oxygen to which it bonds in the product state, and the carbon to which that oxygen is bound. This angle increases slightly from roughly 2.1 to 2.2 radians during the transfer of the proton to His237 and then decreases to the equilibrium value of roughly 1.8 radians when the proton transfer is complete. The bond-breaking and forming distances change in a concerted fashion (Fig. 2F). Note that RC values are dimensionless, and the scale is arbitrary. By convention, negative values denote progress toward the reactant and positive toward the product.

**Fig. 3:**
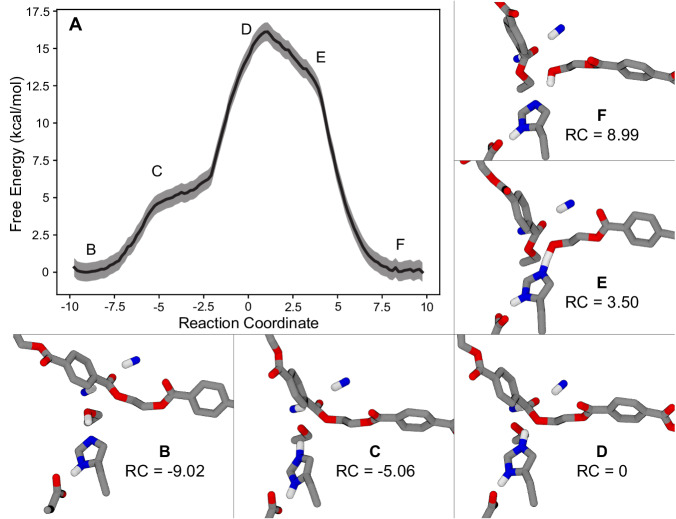
Acylation free energy profile. **A** Free energy profile for the acylation reaction from umbrella sampling along the acylation RC with shaded error bars representing uncertainty estimates from MBAR^100^. Some points of interest are labeled on the plot with corresponding molecular snapshots shown in panels (**B**–**F**), which show a moving histidine mechanism without the transfer of a proton to Asp206. **B** The Michaelis complex corresponds to the left basin, followed by (**C**) an initial hump associated with the transfer of proton from S160 to His237, **D** the transition state at the peak where the MHET carbon adopts a tetrahedral electronic structure, **E** a shoulder where the proton is shared between His237 and the product BHET, and finally **F** the product state with the released BHET and covalent bond between the remaining MHET and Ser160 at the right side of the profile.

The free energy profile of the acylation reaction step was measured using umbrella sampling along the discovered RC (Fig. 3). Although tracking the CVs that constitute the RC suggests a single concerted step, the free energy profile reveals two additional features: an initial shoulder for transfer of the proton from Ser160 to His237, and a second shoulder on the products side as the proton is transferred from His237 to the reaction product BHET. These features emphasize the role that His237 plays in stabilizing proton transfer during the acylation step. The reactive flux correlation function was computed for the acylation RC along this path, yielding the observed value of 1.00 for the transmission coefficient (*κ*) (Fig. 2E)^45–47^. Using the Eyring equation with the free energy of the acylation TS (16.1 ± 0.6 kcal/mol), the transmission coefficient, and 310 K system temperature, the rate constant for the acylation step was estimated to be *k* = 28.8 ± 17.9 s^−1^.

### Elucidating the deacylation reaction coordinate and estimation of reaction rate

To set up the PETase system for QM/MM deacylation simulations, we began with an AEI configuration from the acylation reaction. The BHET product of acylation was removed, and a covalent bond was added between Ser160 and the PET fragment for the purpose of MM equilibration. Following MM and QM/MM equilibration, a water molecule located in the active site was incorporated into the QM region and the system was QM/MM equilibrated once more with DFTB3^42^. The system was then subjected to the same ATESA *find_ts* procedure as described for acylation to locate a putative TS region^43^. AS was again used to generate an ensemble of 19,000 unbiased trajectories, of which approximately 39.6% were reactive.

**Fig. 4:**
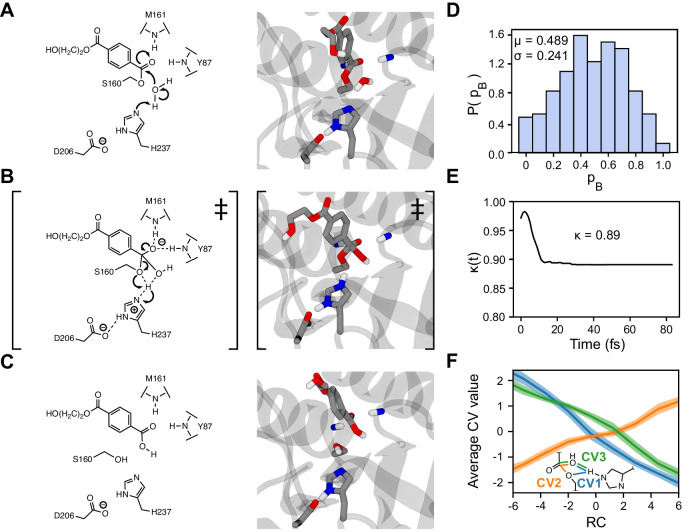
PETase deacylation reaction. **A**–**C** Scheme depicting the steps of the deacylation reaction (*left*) and a corresponding snapshot taken from a reactive AS trajectory (*right*). **A** AEI state, where the catalytic water is poised to attack the acylated PET carboxyl carbon. **B** TS configuration where the catalytic water has attacked the carboxyl carbon of PET, the catalytic water has transferred a proton to His237, the PET carboxyl carbon adopts a tetrahedral geometry, and His237 is midway through the proton shuttle. **C** Product of deacylation, where Ser160 has accepted the proton from His237, the acyl bond between Ser160 and PET is broken, the catalytic triad is restored, and the MHET product has been formed. **D** *p*_B_ histogram obtained from unbiased trajectories initiated from 232 putative TS structures according to the deacylation RC. **E** Reactive flux correlation function was computed using unbiased trajectories initiated from the putative TS region according to the deacylation RC. (**F**) Average collective variable (CV) values were obtained from the ensemble of unbiased trajectories observed during AS. These CVs are CV1 = the difference between the distances Ser160 O–water H and water H–water O; CV2 = the difference between the distances Ser160 O–MHET C and MHET C–water O; and CV3 = the difference between the distances MHET C–water O and water O–water H. All distances in Å. The coordinates from these trajectories were binned according to the value of the deacylation RC from −6 to +6 using 9 bins, wherein the CVs in each bin were averaged, and the standard deviation was taken as an error.

Through inspection of the reactive AS trajectories, illustrated in Fig. 4, the deacylation reaction scheme proceeds through the following steps: (1) from the AEI state, the catalytic water transfers a proton to His237 and attacks the carboxyl carbon of the acylated PET fragment; (2) the PET carboxyl carbon adopts a tetrahedral TS configuration with His237 at the midpoint of the proton shuttle; and (3) the TS collapses, cleaving the scissile ester bond between MHET and Ser160, with Ser160 accepting the proton from His237. The completion of this reaction leaves the system in the product state, with Ser160 regenerated and MHET free to leave the active site.

A total of 182 CVs were screened using iLMax and the trajectory ensemble from AS in the same way as described above for the acylation reaction. As in the acylation step, the optimized RC for the deacylation reaction prominently features the differences between key distances. The selected deacylation RC was $1.55-1.42* {{\rm {CV}}}1+1.39* {{\rm {CV}}}2-0.590* {{\rm {CV}}}3$, based on the CV definitions shown in Fig. 4F and described in the corresponding caption. Just as in the acylation step, the committor histogram (Fig. 4D) was peaked and centered around 0.5, validating the RC found by iLM.

**Fig. 5:**
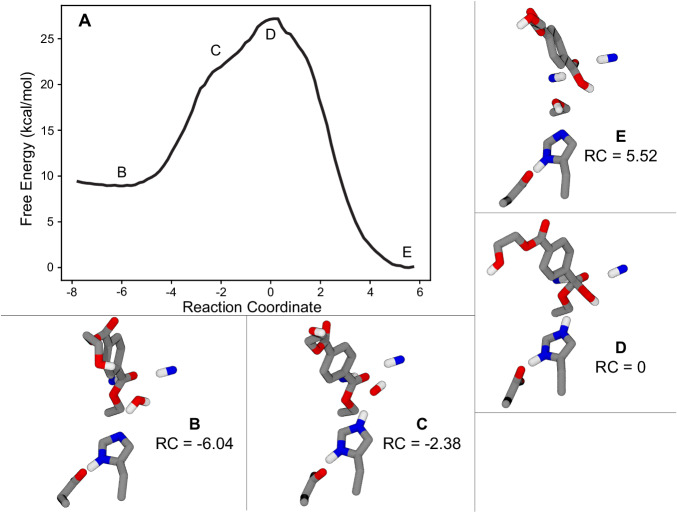
Deacylation free energy surface. **A** Free energy surface for the deacylation reaction, sampled along the deacylation RC. Error bars represent uncertainty estimates from MBAR^100^ and are thinner than the width of the line. The system begins in the AEI state (the deacylation reactants state) with the product BHET from that step removed at the left side (**B**); the first shoulder is associated with the transfer of the attacking water molecule’s excess proton to His237 (**C**); the water then attacks the MHET carbon at the transition state, causing the carbon to adopt a tetrahedral electronic structure (**D**); and finally, Ser160 accepts the proton from His237 and the MHET product is released from its covalent bond with the enzyme (**E**).

The free energy profile of the deacylation reaction step was measured using umbrella sampling along the discovered RC (Fig. 5). The free energy profile has a shoulder on the left side of the transition state for the transfer of the proton from the attacking water to His237, while the transition state occurs when the water attacks the MHET carbon. The reactive flux correlation function was computed for the deacylation RC along this path, yielding the observed value of 0.89 for the transmission coefficient (*κ*) (Fig. 4E)^45–47^. Using the Eyring equation with the free energy of the deacylation TS, the transmission coefficient, and 310 K system temperature, the rate constant for the deacylation step was estimated to be *k* = 0.82 ± 0.10 s^−1^. This rate constant is thus lower than that of the acylation step by about a factor of 35, confirming that deacylation is the rate-limiting step in the overall reaction.

### Investigating the Trp185 conformational change

During QM/MM-based MD equilibration of the AEI to prepare the system for the deacylation reaction, the Trp185 residue spontaneously underwent a conformational change corresponding to the C-C_α_-C_β_-C_γ_ dihedral angle (χ_1C_) changing from −60° to +60°. We observed two distinct stable conformations at the AEI state of the acylation reaction that correspond to the two conformations of Trp185. Based on these observations, we hypothesized that the Trp185 conformational change occurs after the acylation step, potentially altering the conformation of the AEI state for deacylation and lowering the free energy of the AEI state.

**Fig. 6:**
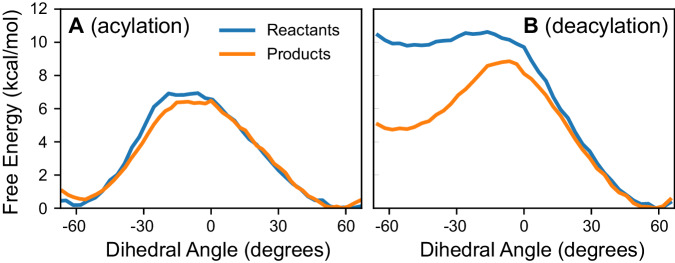
Trp185 dihedral free energy profiles. Free energy surface for rotation along the C-C_α_-C_β_-C_γ_ dihedral angle of Trp185 in the reactant and product states of **A** the acylation step, and **B** the deacylation step. Error bars thinner are than the width of the lines in all cases. The acylation products state and deacylation reactants states differ in that in the latter, the product BHET from acylation has been removed, permitting a reorganization of the active site that greatly favors the +60° basin for Trp185 χ_1C_ dihedral angles. See Fig. 7 for a visualization of Trp185 in each state in the context of the bound substrate.

To investigate this hypothesis, we performed umbrella sampling simulations along the Trp185 χ_1C_ dihedral in both the product and reactant states of the acylation and deacylation reactions. The resulting free energy profiles are shown in Fig. 6. The reactant state occupying the energy basin on the left side of the acylation step (Fig. 6A) corresponds to the binding pose in the prior docking work from which this study was initiated^10^. We observe a very small preference (0.3 ± 0.1 kcal/mol) for the positive-angle state in the products state relative to the reactant state for the acylation reaction, as well as a small decrease in the barrier (roughly 0.4 kcal/mol) to transitions between the two stable conformations.

In contrast, the configuration of the active site for the deacylation step very strongly favors the basin on the right (positive values of the Trp185 dihedral) in both the reactant and product steps (Fig. 6B). After the deacylation reaction takes place, much of this preference is lost compared to the reactants state (a change of 5.0 ± 0.1 kcal/mol), likely owing to the release of the MHET product from Ser160 and its correspondingly increased flexibility. The differential preferences we observe in the Trp185 χ_1C_ dihedral before and after the deacylation reaction may help facilitate product release and inhibit backward reaction progress. Classical MD simulations of the product state were used to monitor the distance between MHET and the Trp185 and Tyr87 residues, as well as the side chain dihedrals of Trp185. As shown in Fig. S2, MHET leaves the active site within 20 ns in 4 of the 10 simulations. Triplicate simulations 200 ns in length of the apo state of PETase from prior work^10^ were analyzed to examine whether the reset of Trp185 χ_1C_ to the −60° basin would occur spontaneously in the absence of any substrate or product. This analysis indicates that the Trp185 χ_1C_ dihedral stays in the +60° basin during apo simulation (Fig. S3).

### Investigating the interactions between Asp206 and His237 of the catalytic triad

Serine hydrolases have been hypothesized to utilize a double-proton transfer mechanism wherein an aspartic acid abstracts a proton from a histidine, allowing histidine to act as a stronger base toward deprotonating the catalytic serine^19,27^. The umbrella sampling trajectories were reused to investigate the role of Asp206 and His237 in the PETase mechanism. We measured the distances between the proton and His237 N_δ1_ and the nearby Asp206 carboxylate O, respectively, as a function of each reaction coordinate. As shown in Fig. S4, Asp206 and His237 maintain a hydrogen bond throughout both reaction steps, and His237 N_δ1_ retains the proton throughout both reaction steps, with the distance only very slightly stretching during regions of each reaction step where another proton is bound to His237’s other nitrogen (N_ε2_). Based on these findings, we predict that PETase does not utilize the double-proton transfer mechanism.

The nature of how His237 transfers the proton from the nucleophile to the leaving group is inherently tied to the interactions with Asp206, wherein the flipping histidine mechanism would require breaking the hydrogen bond between the two, but not so for the moving histidine mechanism^19,26–28^. As depicted in Figs. 2 and 4 for the acylation and deacylation reactions, respectively, a single proton is transferred via His237 N_ε2_ between the nucleophile and leaving groups. Additionally, we measured the side chain dihedrals C-C_α_-C_β_-C_γ_ and C_α_-C_β_-C_γ_-N_δ1_ of His237 (Fig. S5) and found that the dihedrals are somewhat flexible to allow for the shuttling motion of His237 in the proton transfer. However, neither dihedral changes in such a way to suggest that His237 flips, which, taken collectively with the aforementioned hydrogen bonding results, clarify that PETase uses the moving histidine mechanism for proton transfer.

### Examining the oxyanion hole and charge stabilization

Via hydrogen bonding interactions with their backbone amines, the oxyanion hole residues Met161 and Tyr87 have been hypothesized to provide stabilization of charge buildup on the PET carbonyl oxygen^10,21,22,24^. The umbrella sampling simulations were again used to measure the distances from the hydrogens on the backbone nitrogen atoms of Met161 and Tyr87 to the carbonyl oxygen of PET along the RC. As shown in Fig. S6, Tyr87’s backbone amine maintains a hydrogen bond with PET throughout both reaction steps, including the AEI state. Met161’s backbone amine is not hydrogen bonded to PET until Ser160 attacks, at which point the PET carbonyl oxygen moves fully into the oxyanion hole and within the hydrogen bonding distance of Met161’s backbone amine. This hydrogen bonding interaction is maintained until the product state of deacylation. Additionally, we extracted the Mulliken charges at the reactant, product, and transition states of both reaction steps to characterize the charge buildup on the key heavy atoms. As shown in Fig. S7, the carbonyl oxygen of PET initially adopts a charge of roughly −0.7 in both steps, building to −0.9 at the TS of the acylation step. The charge then returns to −0.7 at the AEI and product states for the acylation and deacylation steps, respectively. However, in the transition state of the deacylation step, there is no observed charge buildup on this oxygen, and in fact, the charge on the carbonyl oxygen approaches −0.6. This difference between the charges in the transition states is compensated by the relatively strong negative charge localized on the attacking water oxygen in the deacylation step, compared to the corresponding scissile oxygen in the acylation step, which accumulates relatively little charge during the acylation reaction.

### Revealing the PET and aromatic residue interactions

Guo and coworkers hypothesized that the aromatic ring of PET interacts through an edge-to-face π-π interaction with Trp185 during the reaction, changing to a parallel-displaced π–π interaction at the product state to aid the release of the leaving group^21,25^. To test this hypothesis, we measured the dihedral angle and distances between the planes of aromatic residues and PET in the active site. As depicted in Figs. S8 and S9, the aromatic rings of PET and Trp185 maintain angles and distances consistent with an edge-to-face π-π interaction for the entirety of the acylation step^48^. For the deacylation step, the distance between Trp185 and PET increases slightly, and the angle between them becomes appropriate for a parallel-displaced π–π interaction.

**Fig. 7:**
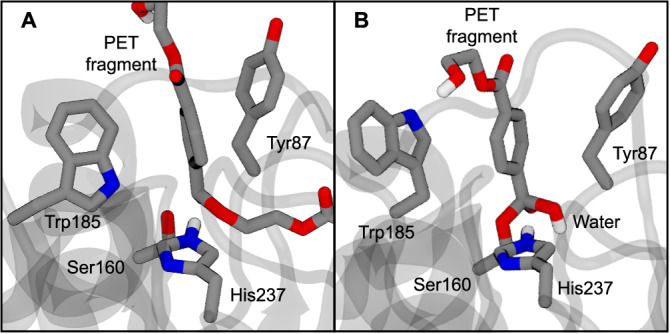
Aromatic interactions of Trp185 and Tyr87. Snapshots of transition states of **A** acylation and **B** deacylation reaction steps highlight aromatic interactions between the PET substrate, Trp185, and Tyr87. Trp185 is in a position corresponding to a negative value of the dihedral shown in Fig. 6 in (**A**) and to a positive value in (**B**).

As shown in Fig. 7, Trp185 interacts with PET though both edge-to-face and T-shaped π–π interactions at different steps, with the distances from Tyr87 and Trp185 to PET more equally spaced after the Trp185 conformational change, and Tyr87 interacting with PET through a parallel-displaced π–π interaction^48^. These residues compose an aromatic clamp observed in PET-degrading cutinases, which may destabilize the substrate, though the effect is not completely understood^22,49^. Here we find that the aromatic stabilization of PET for the acylation step has a little-to-no preference between the Trp185 −60° and +60° basins (Fig. 3). However, for the deacylation step, the change in the aromatic interactions between Tyr87, Trp185, and PET in the Trp185 χ_1C_ + 60° basin appears strongly favorable (Fig. 6B). Overall, these observations are consistent with what Guo and coworkers hypothesized, with the caveat that the parallel-displaced π-π interaction is favored during the deacylation reaction step (as opposed to only during product release).

### Tracking the geometry of the carboxyl carbon

Conflicting hypotheses have been previously presented with regard to whether the tetrahedral configuration belongs to a metastable intermediate or a TS in serine hydrolases^19,27^. We find that the carboxyl carbon of the PET ester undergoes a hybridization state change from *sp*^2^ to *sp*^3^ at the TS and then back to *sp*^2^ for both reaction steps. To characterize this hybridization, we measured the improper dihedral angles of the carboxyl carbon using the same set of umbrella sampling trajectories as described above. The planarity for each transition state is described by two improper dihedrals. For acylation, the first three atoms for each of these impropers were the PET carboxyl carbon (taken as the central atom), the carboxyl oxygen, and the carbon that connects to the adjacent aromatic ring. The fourth atom was either Ser160 O_γ_ or the scissile oxygen of the PET ester. For deacylation, the PET ether oxygen is replaced by the catalytic water oxygen. A planar *sp*^2^ configuration corresponds to an improper dihedral near 0°, while an ideal tetrahedral *sp*^3^ configuration, as measured using the methyl group of alanine residues, corresponds to absolute values of 30–35°. As shown in Fig. S10, the substrate starts in a planar configuration with the ether oxygen and changes to a tetrahedral configuration as it reaches the acylation TS. Correspondingly, the new bond forming via the Ser160 nucleophilic attack also takes on a tetrahedral configuration at the TS. Neither of these improper dihedral angles appears to plateau along the RC, but instead, they simultaneously change complementarily to one another along the RC, with the acylation reaction completing with the acylated ester formed via Ser160 in a planar configuration. A nearly identical scenario occurs for the deacylation reaction. Thus, in both reaction steps, the tetrahedral configuration is predicted to correspond to the TS and not a metastable intermediate.

## Discussion

The mechanism of the two-step PETase reaction was investigated using QM/MM transition path sampling, allowing us to reveal mechanistic details of the reaction without assuming an RC or having a priori knowledge of the TS. We used the *p*_B_ histogram test to validate the RCs obtained *a posteriori* via iLMax, finding that a three-component RC is adequate to describe both reaction steps. Our *p*_B_ histograms fit an ideal Gaussian distribution centered on 0.5, and exceptionally high transmission coefficients demonstrate the advantages of iLMax compared to using the original likelihood maximization method in this case^50–52^. The results we have obtained help answer mechanistic questions posited by studies of other serine hydrolases; particularly the mechanism of proton transfer via His237, hydrogen bonding and charge stabilization in the oxyanion hole, and hydrogen bonding between His237 and Asp206^19,27^. Prior theoretical studies have found that the tetrahedral configuration in the acylation and deacylation reactions can correspond to TS or metastable intermediates, leading to either one or more dominant energetic barriers per reaction step^53–67^. We observe a single TS for each reaction step, coinciding with the tetrahedral configuration, which further supports that both the acylation and deacylation steps proceed in a concerted fashion in the PETase reaction.

Several computational studies have been undertaken to elucidate the mechanism and energetics of ester hydrolysis in *Is*PETase^30–36^. For example, Jerves et al. computed free energy barriers via QM/MM umbrella sampling as a function of the difference in distances for forming and breaking carbon-oxygen bonds, concluding that both acylation and deacylation proceed via a concerted mechanism (without a metastable tetrahedral intermediate), and that acylation is rate-limiting^35^. Boneta et al. follow a similar approach but also include breaking and forming bonds that incorporate the proton transfer, computing free energy surfaces that indicate the existence of a metastable tetrahedral intermediate for both acylation and deacylation, in accordance with the four-step mechanism they assume^34^. Other studies utilize more static calculations, such as ONIOM QM/MM, to calculate reaction energetics for geometries predicted to be part of the reaction pathway^30–33^. A common feature in each of these studies is that the reaction mechanism is assumed a priori, and subsequent energy calculations are performed along an assumed, unverified reaction coordinate. However, accurate determination of the reaction mechanism requires that the mechanism (in the context of reaction rate theory, the “reaction coordinate”^68,69^) be an output of the simulations rather than an input. The approach of Garcia-Meseguer et al., in their comparison of *Is*PETase and FAST-PETase^13^ improves on this approach by utilizing the adaptive string method, allowing the underlying free energy surface to define the minimum free energy path connecting reactants and products, yet the reaction coordinate discovered in this manner still lacks the same degree of mechanistic insight and remains unverified^36^. Computing energetics along unverified reaction coordinates risks missing key features of the free energy surface of the reaction^40^, and therefore cannot be assumed to accurately resolve the important mechanistic questions raised above, such as double proton transfer, moving vs. flipping histidine, whether tetrahedral geometries are metastable intermediates or unstable transition states, etc. Finally, although some of these studies observed the wobbliness of Trp185, they did not account for the motion of Trp185 between reaction steps, which we have shown to be crucially important.

Another common feature of prior simulation work is that the determination of the rate-limiting step is based on activation energies without consideration of kinetic prefactors. Our sampling along the unbiased and verified RCs discovered with transition path sampling finds similar activation barriers of 16.1 and 18.2 kcal/mol for acylation and deacylation steps, respectively, and these RCs then provide for predictions of rate constants based on observed transmission coefficients from unbiased simulations. Likely because of different assumed reaction mechanisms, prior computational work has yielded differing results on the rate-limiting step. Our results indicate that deacylation is rate-limiting with a reaction rate constant estimated as *k* = 0.82 ± 0.10 s^−1^. These results for the reaction rate constant are in good agreement with the experiment. For example, Erickson et al. utilized the Michaelis-Menten relationship to derive a value of *k*_cat_ = 1.5 ± 0.5 and 0.8 ± 0.0 s^−1^ for amorphous PET film and crystalline PET powder, respectively, at 30 °C^70^. Several other relevant kinetics studies of *Is*PETase and related enzymes have been performed at higher temperatures^71–73^.

Our investigation of the PETase reaction mechanism utilized a PET dimer as a substrate analog. PETase has been shown to act on a PET surface, but the molecular nature of this binding is unknown^10,21,22,24,74,75^. We emphasize that there could be differences in chain conformation when bound to a full PET chain and not just a fragment and that the sampled conformations will also be dependent on temperature^76^ and potentially on pretreatment of the PET sample^77^. In addition, enzymes often affect the energetics of reactions they catalyze by altering the distribution of chain conformations in the active site of an enzyme to be different from that in solution, melt, or crystal context. Open questions also remain regarding whether PETase cleaves PET at the chain ends in an exo-fashion or internal chains central to the polymer in an endo-fashion^10^, although recent evidence indicated that a homologous *Thermobifida fusca* cutinase can act via either mode of action, depending on whether the PET substrate is amorphous and mobile (facilitating exo-action), rigid amorphous, or crystalline (with the latter two being mainly degraded by endo-type cuts)^78^. Interestingly, the synergistic MHETase enzyme has also recently been shown to act as an exo-PETase, degrading PET from chain ends^17^. Inherent to these questions is determining the free energy of decrystallization of the PET polymer and whether PETase processes along a chain or unbinds and rebinds to perform the next reaction^79,80^.

**Fig. 8:**
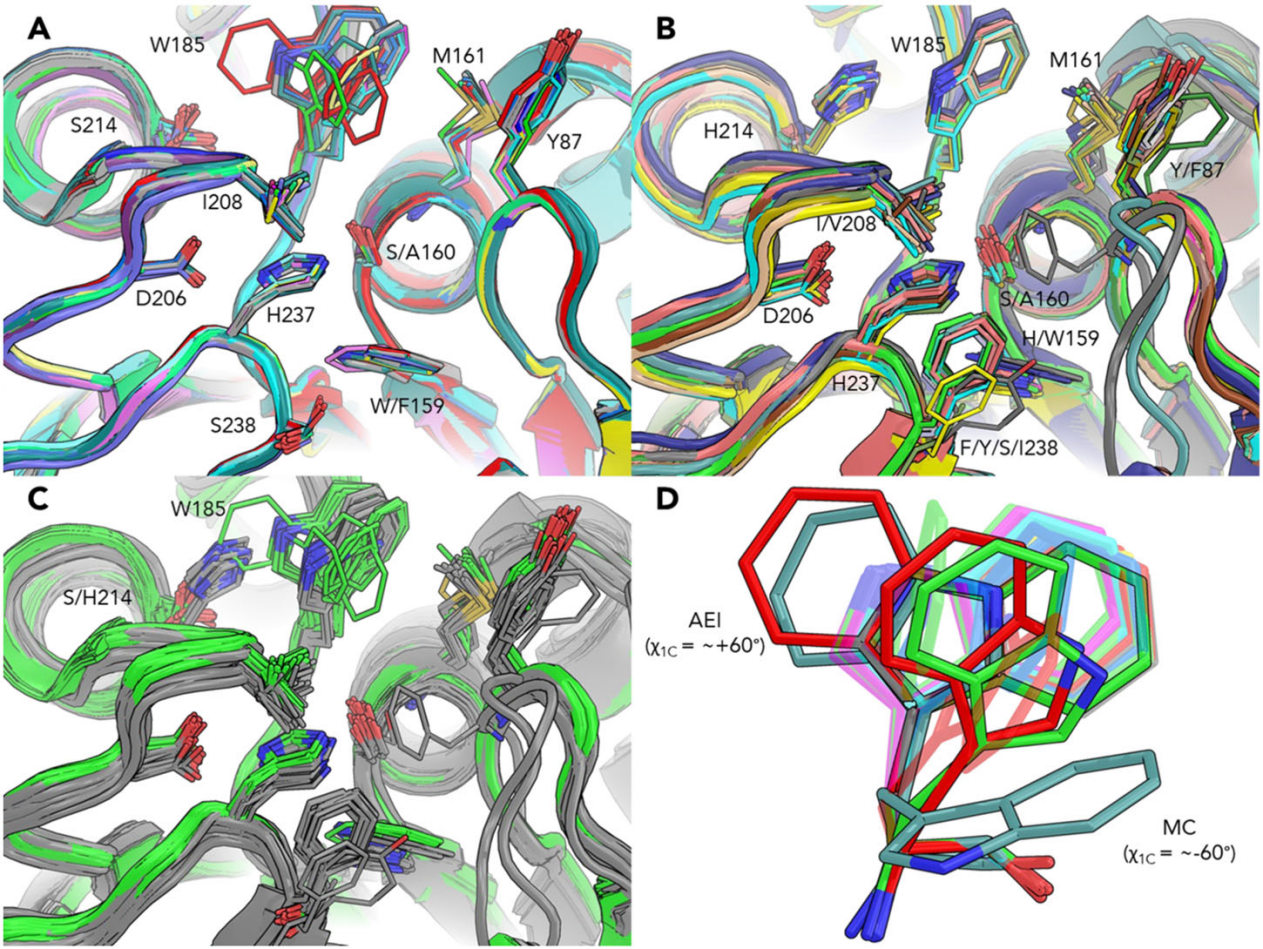
Active site of crystal structures from *I. sakaiensis* PETases and other PET-degrading hydrolases. **A** Superimposition of IsPETase Protein Data Bank (PDB) IDs 6EQE, 6EQF, 6EQG, 6EQH, 5XG0, 5XFY, 5XFZ, 5XH2, 5XH3, 5XJH, 5YNS, 5YFE, 6ANE, 6QCG, 6ILW, and 6ILX^4,10,21,22,24,74,75^. **B** Superimposition of other PET-degrading and cutinase-like hydrolases PDB IDs 4CG1, 4CG2, 4CG3, 5LUI, 5LUJ, 5LUK, 5LUL, 4EB0, 6THS, 6THT, 3VIS, 3WYN, 4WFJ, 5ZNO, 5ZRQ, 5ZRS, 5ZRR, 1JFR, 6AID, 6SBN, and 6SCD^15,49,103–110^. **C** Superimposition of the above structures with IsPETase in green and other PET-degrading hydrolases in gray. Structures in panels **A**–**C** were superimposed using PyMOL with PDB ID 6EQE as the target, and residues were labeled according to our numbering for residues in the same positions. Some crystal structures contained multiple chains, of which all were selected for comparison (27 chains for IsPETase, and 27 chains for other hydrolases), and any active site ligands, solvents, or ions were excluded for clarity. **D** Crystal structure of Trp185 published by Austin et al. (PDB IDs 6EQE, 6EQF, 6EQG, and 6EQH)^10^ in blue, by Han et al. (PDB IDs 5XG0, 5XFY, 5XFZ, 5XH2, and 5XH3)^21^ in green, by Joo et al. (PDB IDs 5XJH and 5YNS)^24^ in cyan, by Liu, B. et al. (PDB ID 5YFE)^74^ in yellow, by Palm et al^4^. (PDB ID 6QCG) in magenta, by Fecker et al. (PDB ID 6ANE)^22^ in red, and by Liu, C. et al. (PDB IDs 6ILW and 6ILX)^75^ in gray. The Trp185 from this study is teal for comparison to the crystal structures, with the Michaelis complex of Trp185 in the χ_1C_ −60° basin in the lower right and in the χ_1C_ + 60° basin of the AEI in the upper left. Trp185 in PDB ID 6ANE chain C (opaque red) was assigned to two orientations that differ from many of the crystal structures, one of which is nearly identical to the one we have shown in the AEI state of the χ_1C_ + 60° basin. The second assignment of the Trp185 position in chain PDB ID 6ANE chain C (second opaque red) matches closely to one of the assigned positions found in PDB ID 5XFZ (opaque green). The Trp185 residues were aligned with PyMOL via atoms C, O, N, C_α_, and C_β_.

A double mutant (S238F/W159H) PETase was generated in prior work to convert the PETase active site into that resembling most cutinases, showing enhanced performance over the wild type towards degrading PET and poly(ethylene furanoate) (PEF)^10^. Docking results from prior work show that binding PET/PEF into the wild type and double mutant PETases each display a different Trp185 binding mode^10^. As we have shown here, this highly flexible residue facilitates the reaction via a reorientation between reaction steps, providing an explanation for the experimentally observed decrease in activity seen in mutations that hinder Trp185 motion, such as S214H^21,25^. Overlaying the reported crystal structures of *I. sakaiensis* PETases with other PET-degrading and cutinase-like hydrolases (Fig. 8) clearly shows steric clashing between some conformations of Trp185 in the *Is*PETase structures with His214 in the other hydrolases (Fig. 8C). Trp185 in this study adopts conformations differing from most of the reported crystal structures of *Is*PETase (Fig. 8D), with the notable exception of chain C in work from Fecker et al., which shows Trp185 in the same conformation we find in the χ_1C_ + 60° basin^22^. A second assignment of the Trp185 position of PETase was found in chain C from Fecker et al. and in the *apo* R103G/S131A mutant by Han et al., which can reasonably be expected to reorient to match the χ_1C_ + 60° configuration during simulation (Fig. 8)^21,22^. While we have shown the reaction initiated with Trp185 in a conformation predicted from our prior induced fit docking work, it is likely that Trp185 facilitates the reaction in the conformation seen in many of the crystal structures. The exact nature of how or if the Trp185 χ_1C_ dihedral resets to the −60° conformation remains an open question. Our study has focused on a small oligomer of PET and the enzyme in solution, where the reset may be a matter of an induced fit effect from a new PET substrate binding in the active site; however, the entirety of the Trp185 motion may be different at the interface of solid PET. Future work with *Is*PETase can provide additional details regarding the role of Trp185 in other conformations during the reaction.

The active sites of *Is*PETase and other PET-degrading and cutinase-like hydrolases bear a striking resemblance, with the catalytic triad, Trp185, and oxyanion residues conserved between these structures. Notable mutations such as S238F and W159H, which resemble many of the cutinase active sites, have been shown to increase the performance of *Is*PETase^10^. Given the similarity between the *Is*PETase and other hydrolase crystal structures (Fig. 8), and the aforementioned mutations, it is likely that the mechanistic insights gained from this study can be applied to the function of PET hydrolysis in these other enzymes.

## Conclusion

Polyester hydrolases derived from microbes hold promise in the biocatalytic deconstruction of PET^7^, though several significant challenges remain to their industrial utilization^8,9,81^. Rational engineering efforts will likely be aided by an enhanced understanding of the hydrolytic mechanism. Here we have studied the PETase reaction mechanism via QM/MM simulations without the use of predetermined RCs or biases, allowing us to elucidate the interactions of the catalytic machinery, active site residues, and substrate throughout both steps of the reaction. For the PETase enzyme, our results show that the moving histidine mechanism is utilized for proton transfer, and Asp206 supports His237 through hydrogen bonding. We have shown that hydrogen bonding in the oxyanion hole stabilizes the PET carboxyl oxygen, with the hydrogen bonds maintained during the AEI state, and that Trp185 and Tyr87 provide π-π stabilization to the PET substrate. Additionally, we have shown that the reorientation of the Trp185 residue between reaction steps facilitates the reaction and that deacylation is the rate-limiting reactive step compared to acylation by a small margin.

PETase is one of many enzymes in the serine hydrolase family, including several that have been demonstrated to hydrolyze PET^1,5–9,82–85^, and the reaction mechanism we have studied here can likely be extended to other enzymes in the family, particularly other PET-degrading cutinases like the ones shown in Fig. 8. The position of the Trp residues analogous to *Is*PETase Trp185 in other PET-degrading cutinases is highly conserved. However, in light of the findings presented here, it may be of interest to protein engineering efforts to study the effect of mutations that modulate the flexibility of this residue in other cutinases.

## Methods

### System preparation

A PETase structure bound to a hydroxyethyl-capped PET tetramer from prior docking work was taken as an initial starting structure^10^. The PET tetramer was reduced to a hydroxyethyl-capped PET dimer by deleting two PET units furthest from the PETase active site, with the ester bond to be cleaved near the center of the dimer. This was done to eliminate any need for QM/MM link atoms in the PET. PET dimer parameters for the CHARMM General Force Field (CGenFF) were obtained from MATCH via the CHARMMing web interface^86–88^. PROPKA 3.1 was used to determine the protonation state of titratable residues for PETase using a pH of 7.0^89,90^. Two disulfide bonds were formed by patching Cys203 to Cys239 and Cys273 to Cys289. The system was solvated with a 15 Å buffer of TIP3 waters in a cubic box (~80 Å edge lengths) with a NaCl concentration of 0.10 M and made electrostatically neutral with excess chloride ions. The resulting system size was composed of 53,816 atoms.

The system was equilibrated classically in CHARMM version c43 using the CHARMM36 protein force field for the protein, CGenFF for the ligand, and the TIP3 water model^86,91,92^. The Langevin thermostat and barostat were utilized with the temperature set to 310 K and pressure set to 1.0 atm, respectively. SHAKE was used to constrain bond lengths involving hydrogen, and periodic boundary conditions were used^93^. A 12 Å cutoff radius was used for short-range interactions, and particle-mesh Ewald (PME) was used for long-range electrostatic interactions^94^. Classical simulations utilized a 2 fs timestep and Leapfrog Verlet integration. The system equilibration was performed in two steps: (1) The protein–ligand complex was held fixed and energy minimized for 500 steps using the adopted basis Newton–Raphson (ABNR) method. This was followed by 1 ns of classical simulation to allow waters and ions to relax about the protein–ligand complex. (2) The protein–ligand complex was released, and the entire system energy was minimized with 500 steps of ABNR minimization. This was followed by 5 ns of classical simulation to relax the entire system.

### QM/MM simulations

Following classical simulations, the CHAMBER utility of ParmEd version 3.0.3 was used to convert the CHARMM coordinate, topology, parameter, and protein structure files to Amber formatted coordinate and topology files for the Sander program of Amber^95,96^. The Amber18 software^97^ was used to carry out all QM/MM calculations with the Density-Functional Tight-Binding semiempirical QM method with third-order expansion^42^ of the DFT total energy (DFTB3) and the parameter set designed for DFTB3 organic and biological applications version 3.1 (3ob-3-1)^98^ to describe the QM region. An 8 Å cutoff was used for short-range, and PME was used for long-range electrostatic interactions^94^. Periodic boundary conditions were used, the timestep was reduced to 1 fs, and SHAKE was applied only to hydrogen in the MM region^93^. The Langevin thermostat and barostat were specified the same for the QM/MM simulations as they were for the MM simulations.

The QM region for the acylation step was composed of the entire PET dimer, side chains of catalytic residues Ser160, His237, Asp206, and side chains of residues Trp159 and Ser238 with hydrogen QM/MM link atoms placed between C_α_ and C_β_. The entire QM region for the acylation step consisted of 104 atoms, including link atoms. The charge of the QM region was set to −1 to account for the protonation state of Asp206. The system was QM/MM energy minimized for a minimum of 2000 steps, then simulated with QM/MM for 200 ps to equilibrate the system. The *find_ts* procedure automated by ATESA^43^ was used to obtain initial putative transition state structures by applying linearly increasing restraints to the atoms with bonds that form or break between the reactant and product states and then selecting intermediate simulation frames and verifying that they produced reactive trajectories using five steps of aimless shooting each (using the aimless shooting method described in the following subsection).

For the deacylation step of the reaction, an AEI configuration was taken from an acylation AS reactive trajectory, and QM/MM was simulated for 100 ps. This system was brought back into CHARMM, where the BHET product was removed, and a patch was written using PET ester parameters to describe the Ser160 O_γ_ to PET carboxyl carbon bond classically. The patch was used to simply satisfy CHARMM. However, the enzyme–ligand complex was held fixed to avoid simulation with these unverified parameters until the system could be made QM/MM again. The enzyme–ligand complex was re-solvated and neutralized, with waters and ions classically minimized and equilibrated stepwise like the acylation step. In this way, we preserve the QM description of the enzyme–ligand configuration between reaction steps. This system was then converted to work with Amber, as described previously.

The QM region for the deacylation step included the acylated PET fragment, side chains of catalytic residues Ser160, His237, Asp206, and side chains of residues Trp159 and Ser238 with hydrogen QM/MM link atoms placed between C_α_ and C_β_. The system was QM/MM energy minimized until reaching the default convergence tolerance and then equilibrated for 200 ps. A nucleophilic water molecule that was naturally present in the active site was incorporated into the QM region, and the system QM/MM equilibrated for another 100 ps. The final QM region consisted of 75 QM atoms, including the hydrogen link atoms. This system was subjected to the same *find_ts* procedure using ATESA as described above for the acylation step^43^.

### Aimless shooting

The flexible-length AS variant of TPS, as automated by ATESA^43^ was used to find the best putative TSs from the ensemble generated from umbrella sampling^40,99^. AS works by assigning random velocities to the atoms of the putative TS based on the Maxwell-Boltzmann distribution of the system temperature. The system is propagated from the putative TS *forward* with the assigned velocities (*V*_0_) as well as *backwards* from the putative TS using the reverse of the assigned velocities (−1*V*_0_). If one direction of the trajectory leads to reactants and the other direction leads to products, then the trajectory is considered reactive^40^. A putative TS that generates a high percentage of reactive trajectories is likely a good TS guess. During the forward portion of the trajectory, a new configuration is saved at a random Δ*t* between 1 and 10 fs, and if the trajectory is reactive, this new configuration is used as the starting point for the next AS step^40^. In this way the configurational space about the TS is thoroughly explored. This AS method does not require any a priori knowledge of the TS configuration; however, initiating AS with a putative TS configuration will reduce the rate of rejected trajectories, and this initial biasing was removed by excluding early trajectories that are collected from analysis by measuring the autocorrelation of each CV and rejecting early trajectories with statistically significant correlations in any of them at the *α* = 0.05 level, as automated by ATESA^40,43^.

For the acylation step, the putative TS configuration was used to initialize 16 independent AS sampling schemes each in both the forward and backward directions, and their results were combined^40^. The reactant basin was defined as: PET fragment C–Ser160 O < 1.8 Å, Ser160 H–Ser160 O > 1.4 Å, Ser160 H–PET fragment O < 1.2 Å, and PET fragment O–PET fragment C > 2.4 Å, while the product basin was defined as PET fragment C–Ser160 O > 2.4 Å, Ser160 H–Ser160 O < 1.2 Å, Ser160 H–PET fragment O > 1.4 Å, and PET fragment O–PET fragment C < 1.8 Å.

For the deacylation step of the reaction, the five putative TS configurations underwent 5 independent AS sampling schemes, each in both the forward and backward directions, and their results were combined^40^. The reactant basin was defined as water O–MHET C < 1.5 Å, water O–water H > 1.2 Å, MHET C–Ser160 O > 1.6 Å, and water H–Ser 160 O < 1.1, and the product basin was defined as water O–MHET C > 1.6 Å, water O–water H < 1.1 Å, MHET C–Ser160 O < 1.5 Å, and water H–Ser 160 O > 1.2.

### Likelihood maximization

Key distances, angles, dihedrals, and differences in distances between reactive atoms, collectively known as CVs, were measured for the AS configurations. Instantaneous rates of change for each CV were also recorded for each configuration. The outcome of forward trajectories, CVs of AS configurations, and CV rates of change were used in an iLMax routine to obtain RCs as a linear combination of some number of CVs according to the *two_line_test* functionality of the likelihood maximization script associated with ATESA^38,39,41,43^. This method automatically identifies the desirable number of CVs to include in the RC and optimizes for the maximum fit to the observed fates of the trajectories of both the CVs and their associated rates of change in order to increase the observed transmission coefficient. Additional details for this method are supplied in the Supplementary Methods.

### Committor probability histogram test

The *p*_B_ histogram test was used to validate the quality of the RCs obtained from iLMax^44^. Given a putative TS configuration, the *p*_B_ is a measure of the likelihood that the TS will evolve to products when assigned random velocities from the Maxwell–Boltzmann distribution^44^. A good TS configuration should exhibit an equal probability of evolving to products as it does reactants. Thus, an ideal *p*_B_ histogram should appear as a Gaussian distribution centered on *p*_B_ = 0.5^44^. For the RCs obtained from iLMax, a value of RC = 0 is associated with the TS. The RC values were computed for the ensemble of all shooting points from AS and a cutoff threshold on the absolute distance of the RC value from zero was selected so as to include roughly 200 configurations. Using ATESA’s *committor_analysis* option^43^, each of these configurations was used as the starting points of 10 simulations using random velocities to initiate each trajectory. Evaluation of trajectory end points used the same basin definitions defined for AS. The fraction of the 10 trajectories that evolved to products was used to construct the *p*_B_ histogram for each RC.

### Potential of mean force

The potentials of mean force (free energy profiles) along the reaction coordinate for each step and for the C-C_α_-C_β_-C_γ_ dihedral angle of Trp185 were evaluated using umbrella sampling, also automated by ATESA^43^. For sampling along the RCs, the RC was divided into evenly spaced windows of width 0.25, in the range [−10, 10] for the acylation step and [−8, 8] for the deacylation step. These ranges were selected arbitrarily so as to extend somewhat past the values of the RC observed at the endpoints of simulations committing in both directions during aimless shooting. Initial configurations for simulations beginning in each window were harvested from reactive trajectories from aimless shooting, and five independent simulations in each window were run for ~20,000 steps each with harmonic restraints of weight 50 kcal/mol-unit^2^, where “unit” is the dimensionless unit of the RC. These simulations were performed using the same simulation settings and QM regions as the aimless shooting simulations. Sampling along the acylation RC was done with ATESA’s pathway restraints option in use to constrain sampling to the observed ensemble of reaction pathways from aimless shooting (see atesa.readthedocs.io for details)^43^. For sampling along the dihedral angles, no QM method was used, and windows in the range [−75, 75]° spaced 5° apart were similarly filled with five independent simulations each. In this case, because initial coordinates in each window were not already available, the restraints increased linearly from 0 to their final value of 50 kcal/mol-degree^2^ over 1000 2-fs steps to equilibrate the coordinates in the appropriate position for the restraints and then run for an additional ~35,000 steps each. The first 1000 steps were omitted before analysis in this case. Potentials of mean force were constructed from umbrella sampling data using the multistate Bennett acceptance ratio (MBAR) as implemented in ATESA using only the subset of the sampling data that had decorrelated from the initial configurations^43,100^.

### Rate constant

To compute the rate constant ($\kappa$) for the reaction requires the transmission coefficient ($\kappa$), the free energy of the TS (∆*G*^‡^), and absolute system temperature, and is computed using the Eyring equation from Transition State Theory:

$$k=\kappa \,({k}_{{\rm {B}}}T/h){{\rm {e}}}^{(-\Delta G^{{{\ddagger}} }/{k}_{{\rm {B}}}T)}$$

where *k*_B_ is the Boltzmann constant, *h* is the Planck constant, and *T* is the absolute temperature of the system^101^. $\kappa$ was calculated from committor analysis simulations as the plateau value of the function:

$$\kappa (t)=\frac{\left\langle \dot{{q}_{0}}* \theta \left(q\left(t\right)\right)\right\rangle }{\frac{1}{2}\left\langle \left|\dot{{q}_{0}}\right|\right\rangle }$$

where *t* is the timestep of the simulation, $q\left(t\right)$ is the value of the reaction coordinate at timestep *t*, $\dot{{q}_{0}}$ is the initial rate of change of the reaction coordinate for a given simulation, $\theta ()$ is the Heaviside step function, and angle brackets indicate the average over each committor analysis trajectory^69^.

### Product state simulations

Ten product state configurations were taken from separate deacylation reactive trajectories. These QM/MM systems were converted from Amber to CHARMM via ParmEd for classical simulation. The CHARMM36 protein force field was used to describe the protein (converting the water hydrogen on Ser160 back to its classical description), CGenFF was used to describe MHET (with the OH from water completing the carboxylic acid), and the TIP3P water model was used. The systems were minimized using 100 steps of ABNR minimization to fix any discrepancies between QM and MM geometries. The systems were made NVT for use with domdec, and briefly heated back to 310 K using a 1 fs timestep for simulation. The thermostat, cutoffs, integrator, and SHAKE were used as described for the purely classical simulations described prior. Coordinates were saved every 2 ps for analysis. Center of mass distances between the six-membered rings of Trp185 and MHET, as well as between Tyr87 and MHET, were measured. The coordinates wrapped from PBCs were unwrapped using the unfold option within the CHARMM merge trajectory command to measure the distance MHET had diffused from the active site.

### Reporting summary

Further information on research design is available in the Nature Portfolio Reporting Summary linked to this article.

### Supplementary information

Supplementary Information

Reporting Summary

## Data Availability

ATESA configurations and other input files, Amber topology files, input and output coordinates for various transition path sampling steps, final aimless shooting results and output files, a text file identifying each of the candidate CVs tracked during aimless shooting and considered during likelihood maximization, and a README file describing all of the above is available free of charge at https://zenodo.org/records/10854763.

## Code availability

The code used to perform all steps in transition path sampling (setup of putative transition states, aimless shooting, inertial likelihood maximization, committor analysis, and umbrella sampling) is available at https://github.com/team-mayes/atesa.
