# Supplementary material for: The reaction mechanism of the Ideonella sakaiensis PETase enzyme
Source: Commun Chem. 2024 Mar 27;7:65. doi: 10.1038/s42004-024-01154-x (PMC10973377; doi:10.1038/s42004-024-01154-x)
Supplement: Supplementary file 1 — Supplementary Information [file 42004_2024_1154_MOESM1_ESM.pdf]

Supplementary Methods

The inertial Likelihood Maximization (iLMax) method[1] was used to obtain RCs as a linear combination of collective variables (CVs) and CV velocities (vCVs) with the form:

$$RC(\boldsymbol{q}, \boldsymbol{q_v}) = \alpha_0 + \sum_{i=1}^{n} (\alpha_i q_i + \alpha_{vi} q_{vi})$$

Where α_0 is a constant, α_i are coefficients of the CVs, α_{vi} are coefficients of the vCVs, q_i are reduced variables of the CVs, and q_{vi} are reduced variables of the vCVs. The q_i and q_{vi} reduced variables have the form:

$$q_i = \frac{CV_i - CV_{i,min}}{CV_{i,max} - CV_{i,min}} \quad ; \quad q_{vi} = \frac{vCV_i - vCV_{i,min}}{vCV_{i,max} - vCV_{i,min}}$$

The original Likelihood Maximization (oLMax) method[2] was used to reweight the iLMax RCs to eliminate velocity dependency, and the resulting RCs were used in further analysis. These oLMax RCs take the form:

$$RC(\boldsymbol{q}) = \alpha_0 + \sum_{i=1}^{n} \alpha_i q_i$$

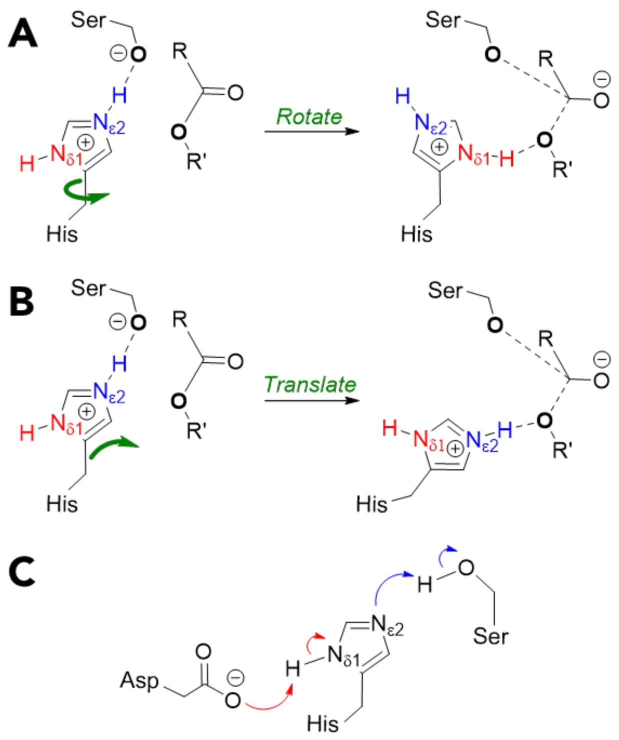

Figure S1: Proton transfer mechanisms. (A) The "flipping histidine" mechanism, where the catalytic histidine accepts a proton from the catalytic serine oxygen at the $N_{\varepsilon 2}$ position, followed by a 180° rotation of the imidazole ring, and delivery of a proton to the leaving group oxygen from the $N_{\delta 1}$ position.[3] (B) The "moving histidine" mechanism, where the proton is transferred from the catalytic serine oxygen to the leaving group oxygen via the $N_{\varepsilon 2}$ atom of the catalytic histidine, facilitated by a translational motion (i.e., left to right in this depiction) of the imidazole ring.[3] (C) The double proton transfer mechanism, otherwise known as the charge relay in serine hydrolases[3], where a catalytic aspartate oxygen accepts a proton from the $N_{\delta 1}$ position of the catalytic histidine first (red), followed by the catalytic histidine accepting a proton at the $N_{\varepsilon 2}$ position from the catalytic serine oxygen second (blue).

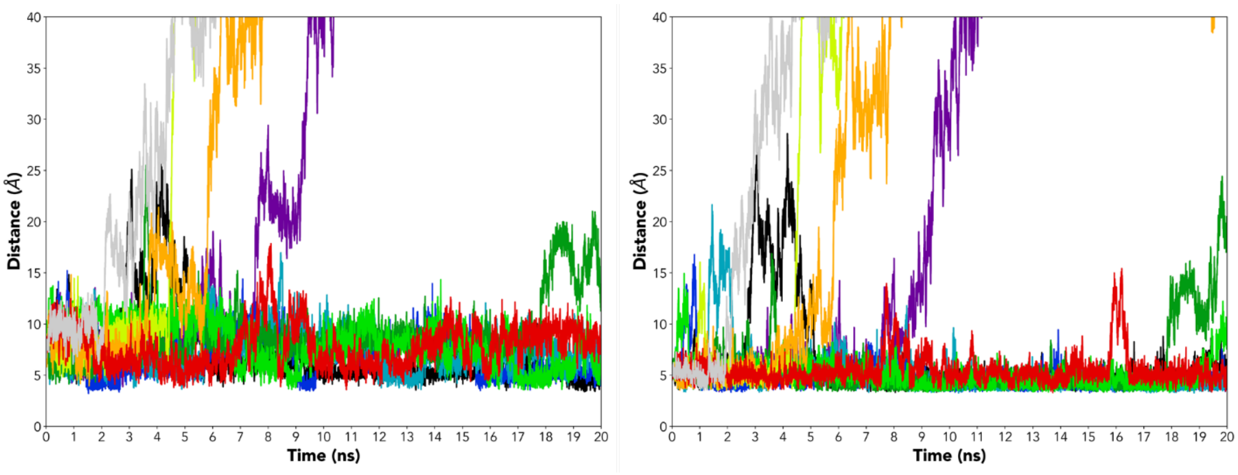

Figure S2: MHET leaving the active site. Center of mass distance of 6C aromatic rings from MHET to Trp185 (left) and Tyr87 (right) to show the MHET product leaving the active site (distance > 15 Å) using 10 unbiased classical simulations starting from the product state of the deacylation reaction. Each simulation was initiated from the last frame of 10 separate reactive trajectories.

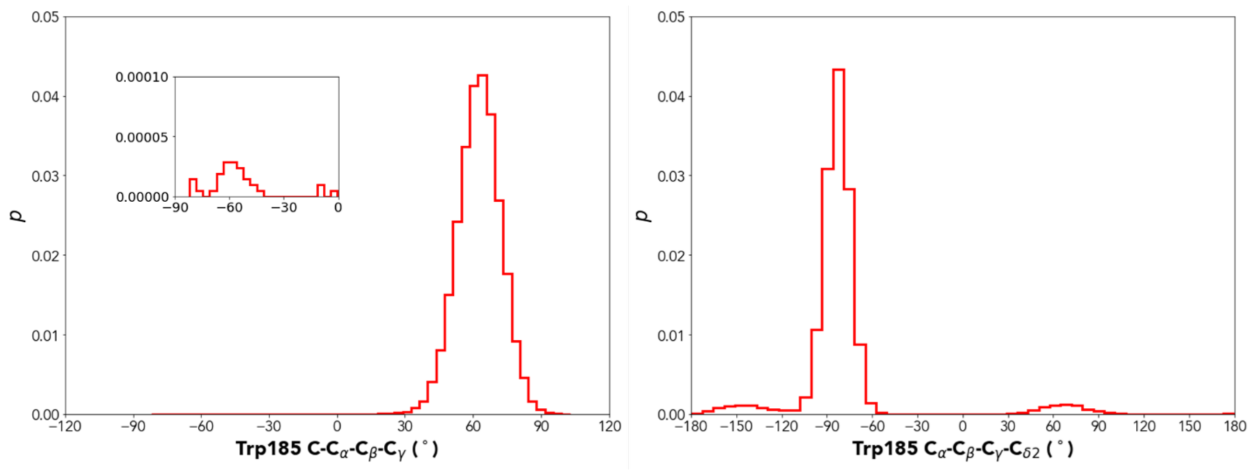

Figure S3: Apo MD of PETase. Histograms of the Trp185 C-C_α-C_β-C_γ dihedral (left) and the Trp185 C_α-C_β-C_γ-$C_{\delta2}$ dihedral (right) from three 200 ns unbiased classical simulations in the apo state from prior work by Austin, *et al.* using 50 bins each.[4] The Trp185 C_α-C_β-C_γ-$C_{\delta2}$ dihedral adopts a few different conformations and it is this dihedral that is most closely associated with the Trp185 "wobble."

Figure S4 through **Figure S10** were generated from the same umbrella sampling trajectories used to produce the free energy profiles for both the acylation and deacylation reactions, except where otherwise noted. In each case we report the average of the respective measurement over each simulation frame, and the standard deviation is used for the error bars.

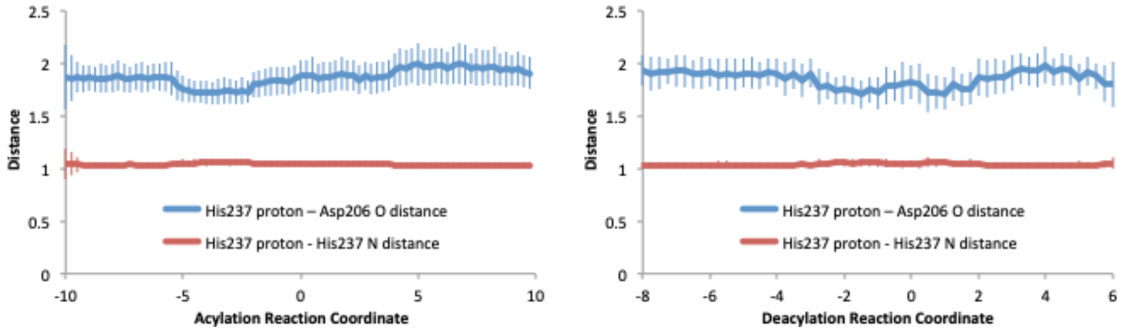

Figure S4: Asp206 to His237 distances. Atomic distances involved in hydrogen bonding between Asp206 and His237 along the RC for the acylation step (left) and deacylation step (right). The Asp206 $O_{\delta2}$ to His237 $H_{\delta1}$ distance is in blue, and His237 $N_{\delta1}$ to His237 $H_{\delta1}$ distance is in red.

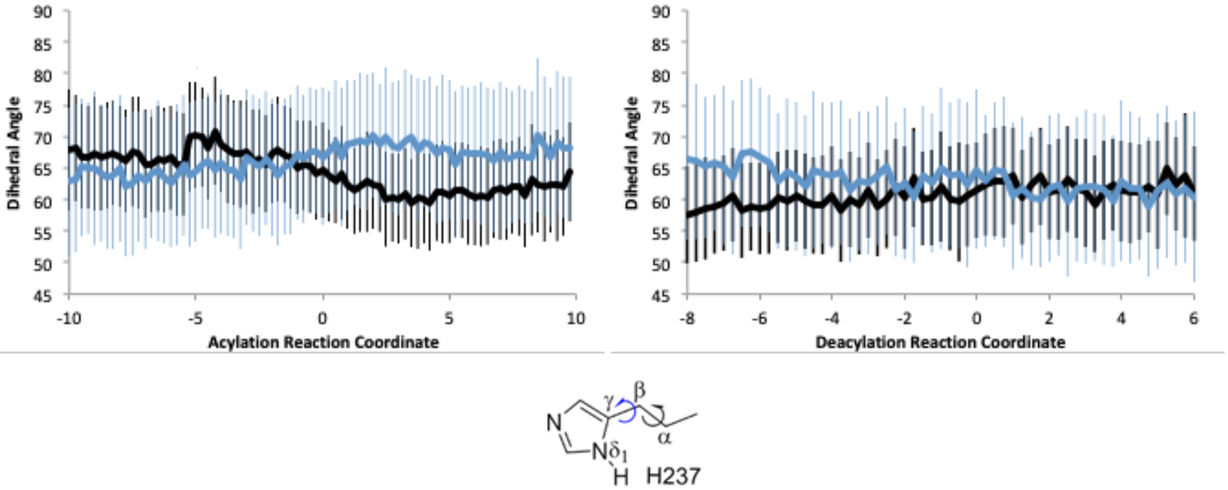

Figure S5: Moving His237 side chain dihedrals. His237 side chain dihedral motion along the RC for the acylation step (left) and deacylation step (right), where the C-C_α-C_β-C_γ dihedral is black, and the C_α-C_β-C_γ-$N_{\delta1}$ dihedral is blue. These motions support the moving histidine mechanism and neither dihedral change in a way to suggest His237 flips.

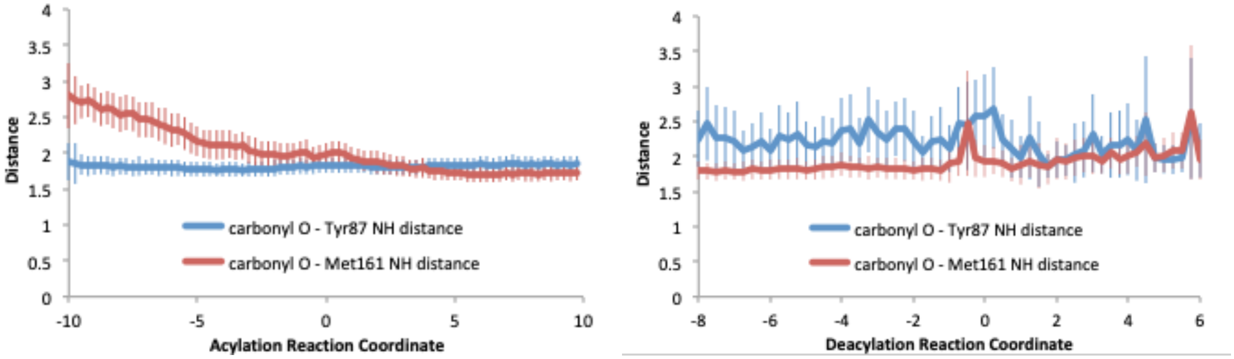

Figure S6: Oxyanion hole hydrogen bonds. Hydrogen bond distances along the RC for the acylation step (left) and deacylation step (right). Met161 bonds more tightly in the intermediate state whereas Tyr87 bonds more tightly in the reactant state.

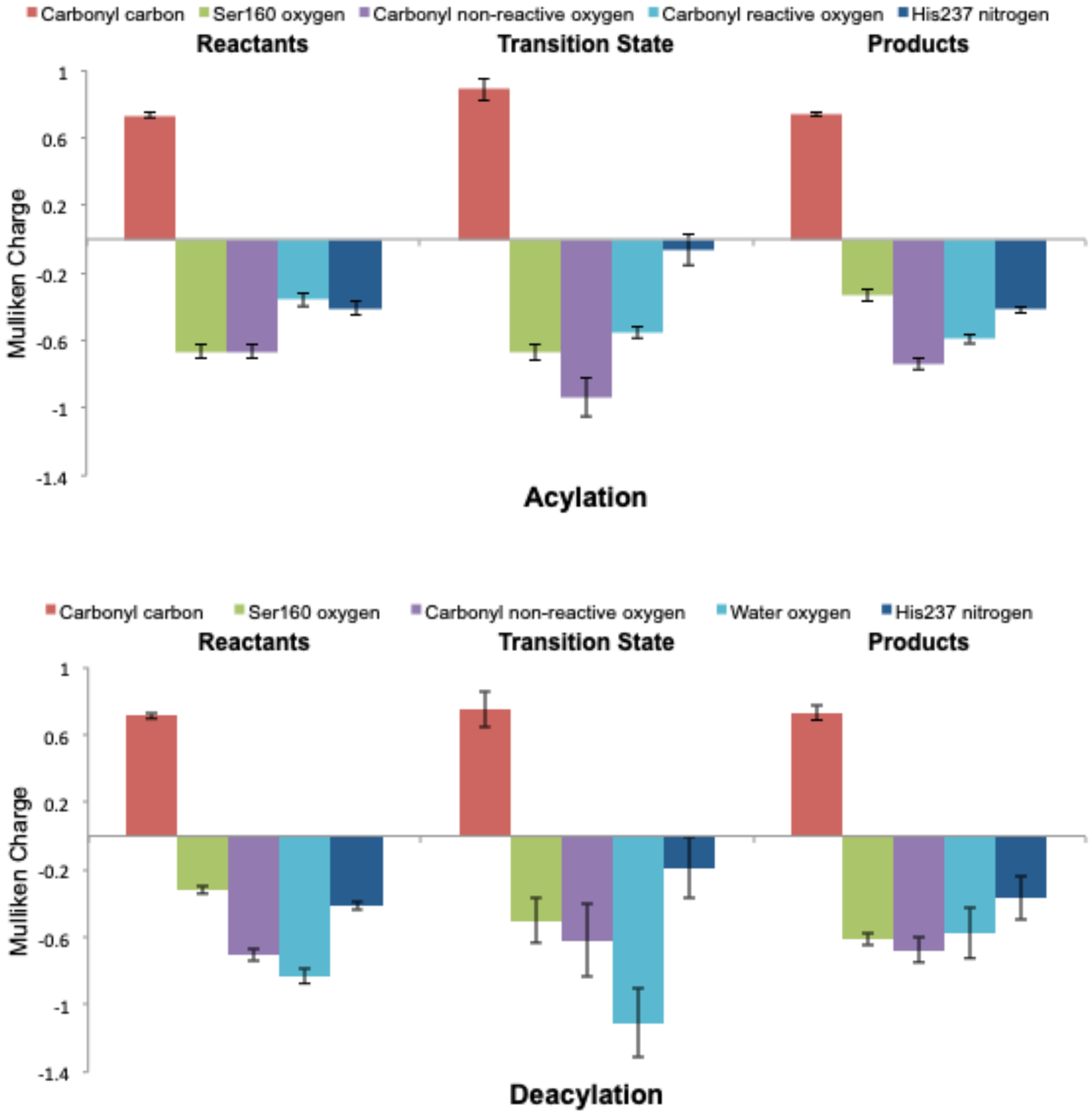

Figure S7: Mulliken charges at the reactive center. Mulliken charges of electronegative heavy atoms in the product, transition, and reactant states for the acylation step (top) and deacylation step (bottom). From left to right, charge on the PET carbonyl carbon is red, the Ser160 O_γ charge is in green, the non-reactive PET carbonyl oxygen is in purple, the reactive oxygen (the PET scissile oxygen in acylation or the catalytic water oxygen in deacylation) is light blue, and His237 $N_{\varepsilon 2}$ is in dark blue. These values were taken from separate repeated umbrella sampling simulations with the same settings as the ones used to produce the free energy profile.

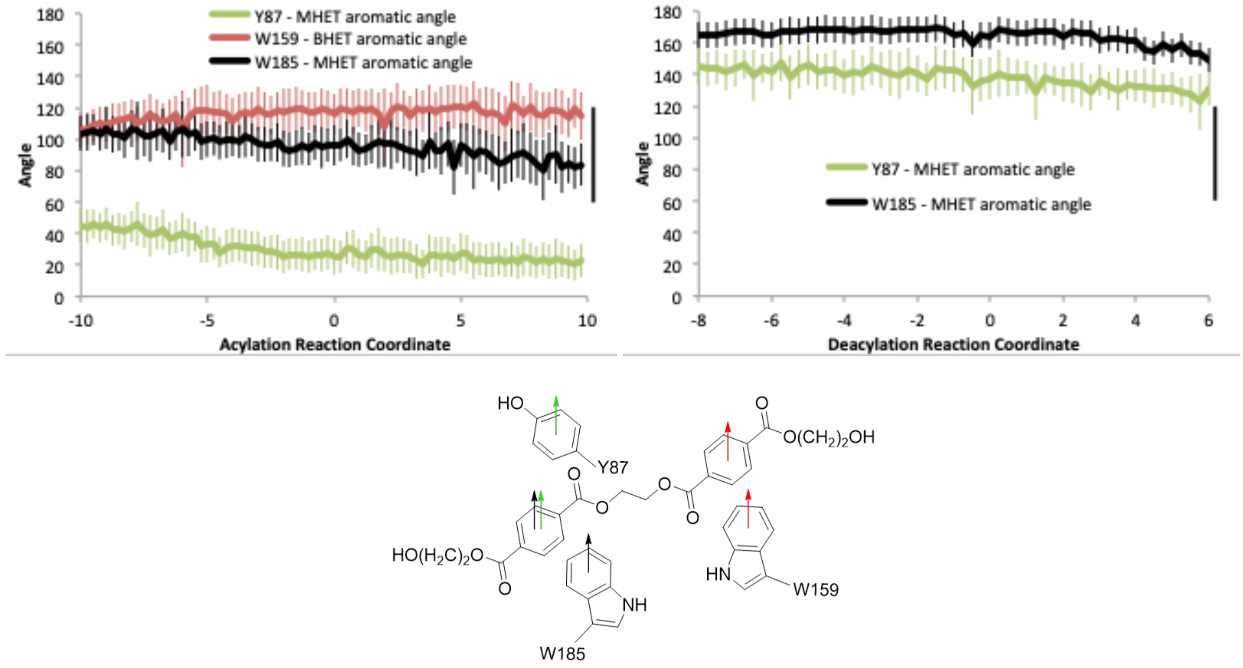

Figure S8: Angles between aromatic rings. Angles between rings of PET and aromatic residues in the active site of PETase for the acylation step (left) and deacylation step (right). The angle measured was between vectors normal to the plane created by the carbons of the aromatic rings of interest. The angle between Trp185 and PET is in black, Tyr87 and PET in green, and Trp159 and PET in red. The Trp159 to PET angle is not applicable to the deacylation step with the removal of the BHET product after the acylation step. In both plots a black line has been added at right to indicate the approximate range of values appropriate for an edge-to-face π-π interaction (about 80-120 degrees) whereas values outside this range are appropriate for a parallel-displaced π-π interaction.

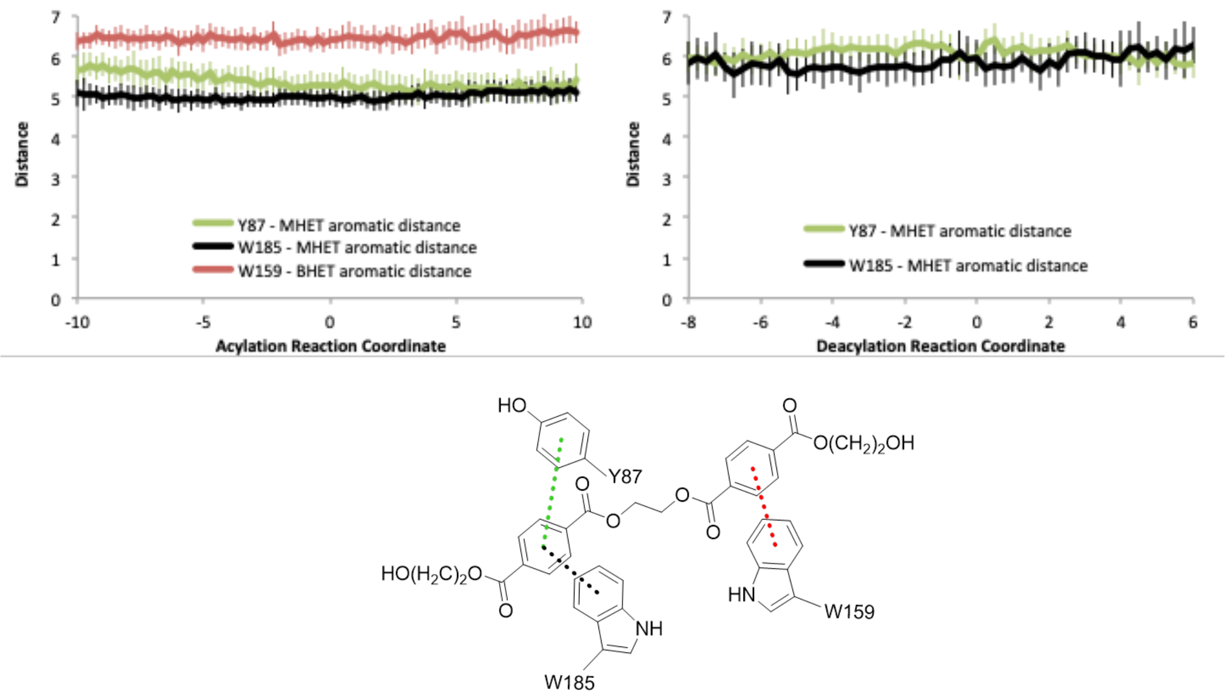

Figure S9: Distances between aromatic rings. Center of mass distances, based on carbon atoms, between aromatic rings of PET and aromatic residues in the active site of PETase for the acylation step (left) and deacylation step (right). The distance between Trp185 and PET is in black, Tyr87 and PET in green, and Trp159 and PET in red. The Trp159 to PET distance was removed for the deacylation step with the removal of the BHET product after the acylation step. The Trp185 C-C_α-C_β-C_γ dihedral change from the -60° basin to +60° basin for the deacylation step changes the Tyr87 and Trp185 aromatic distances to PET for the deacylation step.

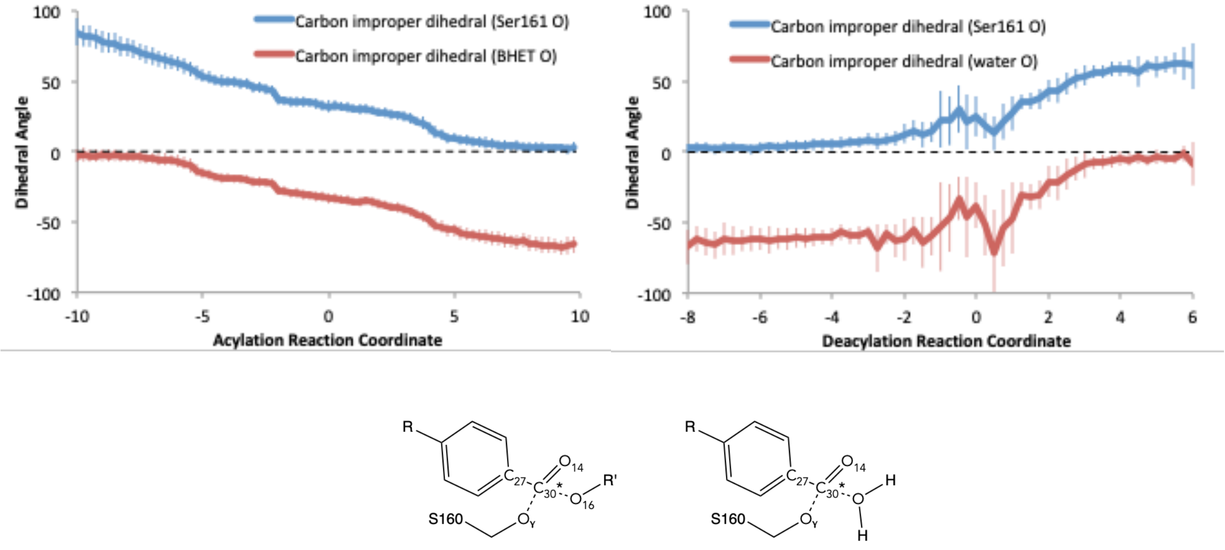

Figure S10: Hybridization of the PET carbonyl carbon. Improper dihedrals centered on the carbonyl carbon of PET for the acylation step (left) and deacylation step (right). The PET C_{30}*-PET O_{14}-PET C_{27}-Ser160 O_γ improper dihedral is in blue for both reaction steps, the PET C_{30}*-PET O_{14}-PET C_{27}-PET O_{16} improper is in red for the acylation step, and the PET C_{30}*-PET O_{14}-PET C_{27}-Water O is in red for the deacylation step. Values close to 0° (black dashed line) correspond to a sp^2 hybridized trigonal planar geometry and absolute values between 30-35° correspond to a sp^3 hybridized tetrahedral geometry. The dip in values just to the right of the transition state in the deacylation plot corresponds to sampling of states where the attacking water has donated a proton to His237 but has been pulled into the bulk water as a hydroxyl ion instead of continuing to attack the carbonyl carbon.

Supplementary References

1. Peters, B. (2012). Inertial likelihood maximization for reaction coordinates with high transmission coefficients. Chem. Phys. Lett. *554*, 248-253.

2. Peters, B., and Trout, B.L. (2006). Obtaining reaction coordinates by likelihood maximization. J. Chem. Phys. *125*, 054108.

3. Hedstrom, L. (2002). Serine protease mechanism and specificity. Chem. Rev. *102*, 4501-4524.

4. Austin, H.P., Allen, M.D., Donohoe, B.S., Rorrer, N.A., Kearns, F.L., Silveira, R.L., Pollard, B.C., Dominick, G., Duman, R., El Omari, K., et al. (2018). Characterization and engineering of a plastic-degrading aromatic polyesterase. Proc. Natl. Acad. Sci. *115*, E4350-E4357.
